# Progesterone treatment reduces neuroinflammation, oxidative stress and brain damage and improves long-term outcomes in a rat model of repeated mild traumatic brain injury

**DOI:** 10.1186/s12974-015-0457-7

**Published:** 2015-12-18

**Authors:** Kyria M. Webster, David K. Wright, Mujun Sun, Bridgette D. Semple, Ezgi Ozturk, Donald G. Stein, Terence J. O’Brien, Sandy R. Shultz

**Affiliations:** Department of Medicine, The Royal Melbourne Hospital, The University of Melbourne, Parkville, VIC 3050 Australia; Anatomy and Neuroscience, The University of Melbourne, Parkville, VIC 3010 Australia; The Florey Institute of Neuroscience and Mental Health, Parkville, VIC 3052 Australia; Department of Emergency Medicine, Emory University, Atlanta, GA 30322 USA

**Keywords:** Concussion, Chronic traumatic encephalopathy, Animal model, DTI, MRI, Treatment, Microglia, Macrophages, Astrogliosis, Lipid peroxidation

## Abstract

**Background:**

Repeated mild traumatic brain injuries, such as concussions, may result in cumulative brain damage, neurodegeneration and other chronic neurological impairments. There are currently no clinically available treatment options known to prevent these consequences. However, growing evidence implicates neuroinflammation and oxidative stress in the pathogenesis of repetitive mild brain injuries; thus, these may represent potential therapeutic targets. Progesterone has been demonstrated to have potent anti-inflammatory and anti-oxidant properties after brain insult; therefore, here, we examined progesterone treatment in rats given repetitive mild brain injuries via the repeated mild fluid percussion injury model.

**Methods:**

Male Long-Evans rats were assigned into four groups: sham injury + vehicle treatment, sham injury + progesterone treatment (8 mg/kg/day), repeated mild fluid percussion injuries + vehicle treatment, and repeated mild fluid percussion injuries + progesterone treatment. Rats were administered a total of three injuries, with each injury separated by 5 days. Treatment was initiated 1 h after the first injury, then administered daily for a total of 15 days. Rats underwent behavioural testing at 12-weeks post-treatment to assess cognition, motor function, anxiety and depression. Brains were then dissected for analysis of markers for neuroinflammation and oxidative stress. Ex vivo MRI was conducted in order to examine structural brain damage and white matter integrity.

**Results:**

Repeated mild fluid percussion injuries + progesterone treatment rats showed significantly reduced cognitive and sensorimotor deficits compared to their vehicle-treated counterparts at 12-weeks post-treatment. Progesterone treatment significantly attenuated markers of neuroinflammation and oxidative stress in rats given repeated mild fluid percussion injuries, with concomitant reductions in grey and white matter damage as indicated by MRI.

**Conclusions:**

These findings implicate neuroinflammation and oxidative stress in the pathophysiological aftermath of mild brain injuries and suggest that progesterone may be a viable treatment option to mitigate these effects and their detrimental consequences.

## Background

Mild traumatic brain injuries (mTBI), including concussions, are a common medical problem worldwide [1]. mTBI is defined as a complex pathophysiological process induced by traumatic biomechanical forces to the brain and often occurs in motor vehicle accidents, assaults, slips, and falls and in combative sports and military settings [1]. Although a patient will typically recover within hours to weeks after a single mTBI [1, 2], there is growing evidence that repeated mTBI (rmTBI) can have cumulative and persisting neurological consequences. For example, rmTBI has been associated with chronic cognitive impairments and an increased risk of depression [3–5]. Furthermore, rmTBI has been linked with a number of neurodegenerative conditions, such as chronic traumatic encephalopathy (CTE), and is implicated in connection with severe cognitive, emotional and motor abnormalities [6].

Although a number of pathophysiological mechanisms have been postulated to contribute to the cumulative and chronic effects of rmTBI, there is growing evidence suggesting important roles for neuroinflammation and oxidative stress [7, 8]. Neuroinflammation is common in TBI patients, as well as in other neurodegenerative conditions associated with mTBI, including Alzheimer’s disease and motor neuron disease [8, 9]. Furthermore, both our laboratory and others have observed elevated and persisting neuroinflammation and oxidative stress in experimental models of mTBI, which coincides with progressive brain damage and chronic behavioural abnormalities [7, 10–17].

To date, there is no clinically available intervention known to prevent the cumulative and chronic consequences of rmTBI. However, considering the possible role of neuroinflammation and oxidative stress in the pathogenesis of rmTBI, an intervention that targets these secondary injury pathways may have therapeutic effects. Progesterone (PROG), a neurosteroid originally named due to its progestational role during pregnancy, exerts neuroprotective effects in experimental brain insults, including TBI [18–22]. Of particular relevance here, PROG is safe for clinical use and has been demonstrated to have potent anti-inflammatory and anti-oxidant properties [18, 21, 22].

Considering the high incidence of mTBI, the cumulative and chronic consequences of rmTBI, the lack of an effective treatment strategy, as well as the likely pathological involvement of neuroinflammation and oxidative stress in the aftermath of rmTBI, we here investigated the use of PROG treatment in a rat model of rmTBI. With the hypothesis that PROG treatment would ameliorate long-term behavioural impairments via the attenuation of post-traumatic inflammation and oxidative stress, rmTBI and sham-operated rats were treated with PROG or vehicle control and assessed after a 12-week post-treatment recovery period. We herein report that PROG treatment in rmTBI significantly reduced neuroinflammation, oxidative stress, brain damage and cognitive and motor impairments.

## Methods

### Animals

Fifty young adult male Long-Evans hooded rats were obtained from the animal breeding service of the Melbourne Brain Centre and were used as subjects. All rats were 8–12 weeks of age, weighed 250–300 g, and were experimentally naïve prior to surgical procedures. After surgery, all rats were housed individually under a 12 h:12 h light/dark cycle with ad libitum access to food and water. All procedures were performed in accordance with the guidelines of the Australian Code of Practice for the Care and Use of Animals for Scientific Purposes written by the Australian National Health and Medical Research Council and were approved by the University of Melbourne Animal Ethics Committee (#1112173).

### Experimental groups and treatment

Rats were assigned into two injury groups: repeated mild fluid percussion injury (rFPI) or sham injury (SHAM). Rats were then further assigned to two treatment groups: cyclodextrin 22.5 % vehicle treatment (VEH; Sigma, Sydney, Australia) or PROG treatment (8 mg/kg in 22.5 % cyclodextrin; Sigma, Sydney, Australia). Thus, there were a total of four experimental groups: SHAM + VEH, SHAM + PROG, rFPI + VEH, and rFPI + PROG.

After each injury, the rat received an intraperitoneal injection of their assigned treatment at 1 h post-injury. This was followed by a daily subcutaneous injection of their assigned treatment for 5 days. This occurred after each of their three injuries. The dose and treatment regimen was based on previous studies reporting neuroprotective effects with PROG treatment after brain injury [18–22].

### Injury model

Previous studies report that a single mild fluid percussion injury (FPI) in the rat induces transient behavioural and pathophysiological changes that occur in the absence of significant neuronal loss or structural brain damage [23–25], which is consistent with what may occur after a single mTBI in humans [1]. Furthermore, our laboratory [10, 11] and others [12, 24] have found that rFPI results in cumulative and long-term neurological changes in rats that bear resemblance to those reported in humans who have suffered rmTBI and CTE.

All of the surgical and rFPI procedures used in this study were based on standard protocols as previously described and used by our group [10, 11]. Briefly, rats were placed in a sealed Plexiglas box into which 4 % isoflurane and 2 L/min oxygen flow was introduced for anaesthesia. Rats were then placed in a stereotaxic frame via ear bars, with anaesthesia maintained at 2 % isoflurane and 1 L/min oxygen, and given a subcutaneous injection of analgesic (carprofen 5 mg/kg). A craniotomy (5 mm diameter) was performed on all rats, centred over the following coordinates with reference to the bregma: anterior/posterior −3.0 mm, medial/lateral 4.0 mm. A hollow plastic injury cap was sealed over the craniotomy, and a removable plug was inserted into the injury cap to seal the craniotomy at all times except during mild FPI or sham injury. Immediately after the surgery, rats were attached to the FPI device. At the first response of a hind limb withdrawal to a toe pinch, rats assigned to the rFPI groups were administered a mild FPI that was induced by a 1–1.5 atm fluid pulse to the brain. Rats from the SHAM groups were administered a sham injury, which involved identical procedures as those for a FPI except that the fluid pulse was not administered. Two subsequent injuries were performed in the same manner at 5-day intervals. Apnea, return of toe pinch reflex, and self-righting reflexes were monitored after each of the injuries as indicators of injury severity [10, 11, 23] (Table 1).

**Table 1:** Acute post-injury outcomes

	Injury 1		Injury 2		Injury 3	
	Hind limb	Self-righting	Hind limb	Self-righting	Hind limb	Self-righting
SHAM + VEH	0 ± 0	98.9 ± 5.2	0 ± 0	98.1 ± 6.5	0 ± 0	93.3 ± 5.0
SHAM + PROG	0 ± 0	101.8 ± 5.8	0 ± 0	99.2 ± 6.4	0 ± 0	96.1 ± 7.4
rFPI + VEH	0.83 ± .56*	149.2 ± 13.4*	2.08 ± .96*	159.9 ± 13.7*	1.25 ± .90*	160.2 ± 9.6*
rFPI + PROG	1.15 ± .61*	158.7 ± 13.0*	1.54 ± .67*	150.2 ± 10.5*	1.54 ± .87*	154.5 ± 8.0*

Regardless of the assigned treatment, rFPI resulted in worse acute injury severity outcomes as indicated by increased hind limb withdrawal and self-righting reflex times compared to the SHAM groups. There was no apnea observed after any of the injuries

*rFPI significantly greater than sham-injured groups, *p* < 0.001

### Behavioural testing

Behavioural testing began 12 weeks after the final day of treatment. Testing was carried out over five consecutive days and was completed by a researcher blinded to experimental conditions on all 50 rats. Rats first underwent testing in the elevated plus maze to assess anxiety-like behaviours as previously described [10, 11, 26, 27]. Briefly, rats were placed into the maze facing an open arm and allowed 5 min to freely explore the maze. An overhead camera recorded behaviours, and the number of entries into and the amount of time spent in open or closed arms were quantified by *EthoVision®* Tracking Software (Noldus™, Netherlands). A percentage score was calculated for the time spent in the open arm, as this is decreased in rats experiencing heightened anxiety [26, 27]. Entries into the closed arm were calculated as a measure of locomotion and were defined by all four paws having entered the arm [11].

Rats were next tested in the open field to assess locomotor and anxiety-like behaviours as previously described [10, 11, 23]. Briefly, the rats were individually placed in a circular open arena (100 cm diameter, 20 cm high wall) and allowed to freely explore for 10 min. An overhead video camera recorded behaviour, and *EthoVision*® software was used to calculate the total distance travelled (cm), as well as the time spent and number of entries into the inner area (50 cm diameter) of the open field.

To assess cognition, water maze testing was conducted as previously described [27]. A circular pool (163 cm diameter) was filled with tap water (29 °C), and a hidden acrylic escape platform (10 cm diameter) was submerged 2 cm below the water surface in one of the quadrants of the pool. Water maze testing involved an acquisition session (day 1) and a reversal session (day 2), with each day consisting of ten trials with a maximum time of 60 s given for each trial. Each trial began at one of the four locations in the pool (north, south, east or west) that were pseudo-randomized to prevent sequential starts from the same location. A trial began when a rat was placed in the pool next to and facing the pool wall and ended when either the rat reached and stood on the hidden platform or 60 s elapsed, at which point the experimenter would guide the rat onto the platform. An overhead video camera recorded behaviour and *EthoVision*® software used to calculate the search time to locate the platform and the number of direct and circle swims as measures of spatial place memory [27]. Swim speed (cm/s) was also calculated as a measure of motor ability. All the settings for the acquisition session and reversal session were the same, with the exception of the escape platform, which was moved to the opposite quadrant [11, 28].

The beam task was used to assess sensorimotor function as previously described [27, 28]. During beam training, rats were given five trials to traverse a 100-cm-long beam with a width of 4 cm and an additional five trials to traverse a 100-cm-long beam with a width of 2 cm. Beam testing occurred 24 h after beam training and consisted of ten trials on the 2-cm-wide, 100-cm-long beam. A maximum of 60 s was permitted for each trial. Traverse times and the number of slips and falls were scored [10]. Rats that fell off the beam were scored with the maximum time.

Depression-like behaviour was assessed using the forced swim task [12, 29]. Rats underwent forced swim training on day 4 of behavioural testing. For training, the rat was placed in the forced swim apparatus, which consisted of a clear cylinder (diameter = 30 cm, height = 40 cm) filled to a depth of 30 cm with water at 25 °C for 15 min. Forced swim testing occurred 24 h after forced swim training. Each rat was placed in the apparatus for 5 min. Behaviour was recorded by a video camera from a horizontal angle and later scored to calculate the time each rat spent immobile, the time spent escaping and the time spent swimming. Only behaviours that persisted longer than 2 s were scored.

### Brain tissue preparation

One day after completion of behavioural testing, rats were killed to obtain either fresh brain tissue for measurement of lipid peroxidation (*n* = 5/group) or fixed brain tissue for MRI and immunofluorescence analyses (*n* = 7–8/group). For fresh tissue collection, rats were deeply anaesthetised and decapitated, and the ipsilateral cortex was rapidly dissected. Fresh tissue was then immediately frozen in liquid nitrogen and later stored at −80°C. For fixed brain tissue collection, rats were deeply anaesthetised and transcardially perfused with ice-cold phosphate-buffered saline followed by 4 % paraformaldehyde in phosphate-buffered saline. Whole brains were removed and stored in 4 % paraformaldehyde at 4 °C until MRI scanning. After MRI, fixed brains were embedded in paraffin for immunofluorescence procedures [23, 30, 31]. All analyses were conducted by investigators blinded to injury or treatment group allocation.

### MRI data acquisition

A 4.7 T Bruker Avance III scanner (Bruker™ Biospec®, USA) with a 30-cm horizontal bore was used for MRI scanning. The magnet was fitted with a BGA12S2 gradient set and actively decoupled volume transmit and four-channel surface receive coils for imaging. Ex vivo brains were placed in 15-ml falcon tubes filled with paraformaldehyde and taped to the surface coil to be scanned.

The protocol for scanning consisted of a three-plane localizer sequence followed by multi-slice axial, coronal and sagittal images to accurately determine the orientation of the rat brain. A rapid acquisition with relaxation enhancement (RARE) sequence was used to acquire T_2_-weighted images with the following imaging parameters: RARE factor = 8, effective echo time = 36 ms, repetition time = 12 s, matrix size = 144 × 72, field of view = 28.8 × 14.4 mm^2^, number of axial slices = 60, isotropic spatial resolution = 200 × 200 × 200 μm^3^, and number of excitations = 32.

A 3D echo planar sequence was used to perform diffusion-weighted imaging with the following parameters: repetition time = 2500 ms, field of view = 32 × 16 × 12 mm^3^, echo time = 62 ms, matrix size = 128 × 64 × 48, and isotropic resolution = 250 × 250 × 250 μm^3^. Diffusion weighting was performed with the diffusion duration (*δ*) = 7 ms, the diffusion gradient separation (Δ) = 20 ms, and *b* value = 8000 s/mm^2^ in 81 non-collinear directions with 9 non-diffusion images (*b*_0_).

### MRI analysis

MRI analysis was completed by two researchers, both of whom were blinded to the experimental conditions. As previously described [27, 31, 32], one researcher used FSL View (Analysis group, Oxford, UK) to manually trace eight a priori regions of interest (ROIs) on T_2_-weighted images. ROIs included the ipsilateral and contralateral hippocampus, cortex, corpus callosum and lateral ventricles. ROIs were traced on consecutive coronal MRI slices, and only slices containing hippocampus were analysed. The second researcher confirmed the accuracy of the ROI traces and processed the MRI data. The total volume for each ROI was calculated using MATLAB® (The MathWorks®, Natick, MA, USA).

Diffusion tensor imaging (DTI) analysis was conducted in order to assess the integrity of the corpus callosum, a major white matter tract commonly affected by TBI [27, 32]. ROI masks of the corpus callosum were transformed into the diffusion image space using Advanced Normalization Tools (ANTs, http://stnava.github.io/ANTs/), and mean DTI measures including fractional anisotropy (FA), mean diffusivity, radial diffusivity and axial diffusivity were calculated using the FSL diffusion toolbox (FDT, http://fsl.fmrib.ox.ac.uk/fsl/fslwiki/FDT) and MATLAB® software.

### Immunofluorescence analysis

As previously described [31–33], immunofluorescence staining and analysis was performed to assess neuroinflammation. Three 4-μm coronal sections from each rat were selected and obtained at the level of injury (−3.0 mm bregma). The sections were deparaffinized and rehydrated [31, 33, 34], then immersed in 0.21 % citric acid buffer (pH 6.0) at boiling temperature in a 1250-W microwave oven for 15 min for antigen retrieval. The sections were then permeabilized with 0.1 % Triton X-100 in phosphate-buffered saline for 10 min and covered in 5 % bovine serum albumin for 60 min at room temperature, followed by incubation with primary antibodies overnight at 4 °C [31, 33]. Anti-GFAP antibody (1:500, rabbit Dako®, Carpentaria, CA, USA) was used as a marker for reactive astrocytes, which have increased expression of GFAP, enlarged cytoplasm and bear elongated and hypertrophic processes [35]. Anti-CD68 (1:500, mouse Abcam®, Cambridge, UK) antibody was used as a marker for activated microglia/macrophages with an amoeboid morphology/phagocytic function [36–38]. The sections were washed in PBS then incubated for 60 min with goat anti-rabbit secondary antibody (1:800, Alexafluor® 488, USA), for GFAP staining, or goat anti-mouse secondary antibody (1:500, Alexafluor® 488, USA), for CD68 staining. The sections were washed again and incubated at room temperature with Sudan Black for 10 min. Slides were then dehydrated and coverslipped [31, 33].

For analysis, photomicrographs of GFAP and CD68 staining were captured using a Carl Zeiss® fluorescence Axioplan-2 microscope at ×20 magnification, under fixed exposure times and light illumination settings to ensure objectivity in image quality and position. One photomicrograph of the most immunoreactive position in the ipsilateral cortex for a total of three photomicrographs for each rat was captured, for a total of three photomicrographs per rat.

GFAP was quantified using the threshold function on ImageJ software (NIH, USA) to create a semi-quantitative index of immunoreactivity by summing the immunopositive area within the digital image, expressed in square micrometers [31, 33]. CD68-positive cells were manually counted on each photomicrograph, and the numbers from the three sections were summed for each rat [31, 33].

### Measurement of lipid peroxidation

The relative levels of malondialdehyde (MDA), an indicator of lipid peroxidation, were measured using a thiobarbituric acid reactive substances (TBARS) assay as previously described [11, 28]. Tissue samples were prepared as directed from the MDA assay kit (MAK085; Sigma-Aldrich®, St Louis, MO, USA).

### Statistical analysis

SPSS 21.0 was used for all statistical analyses. Water maze search time was analysed using a repeated measures analysis of variance (ANOVA) with injury and treatment as between-subjects factors and trial as the within-subjects factors. Two-way ANOVAs, with injury and treatment as between-subjects factors, were used for all other analyses. Bonferonni post hoc comparisons were used when appropriate. Statistical significance was set at *p* ≤ 0.05.

## Results

### PROG treatment reduces cognitive and sensorimotor deficits in rFPI

**Fig. 1:**
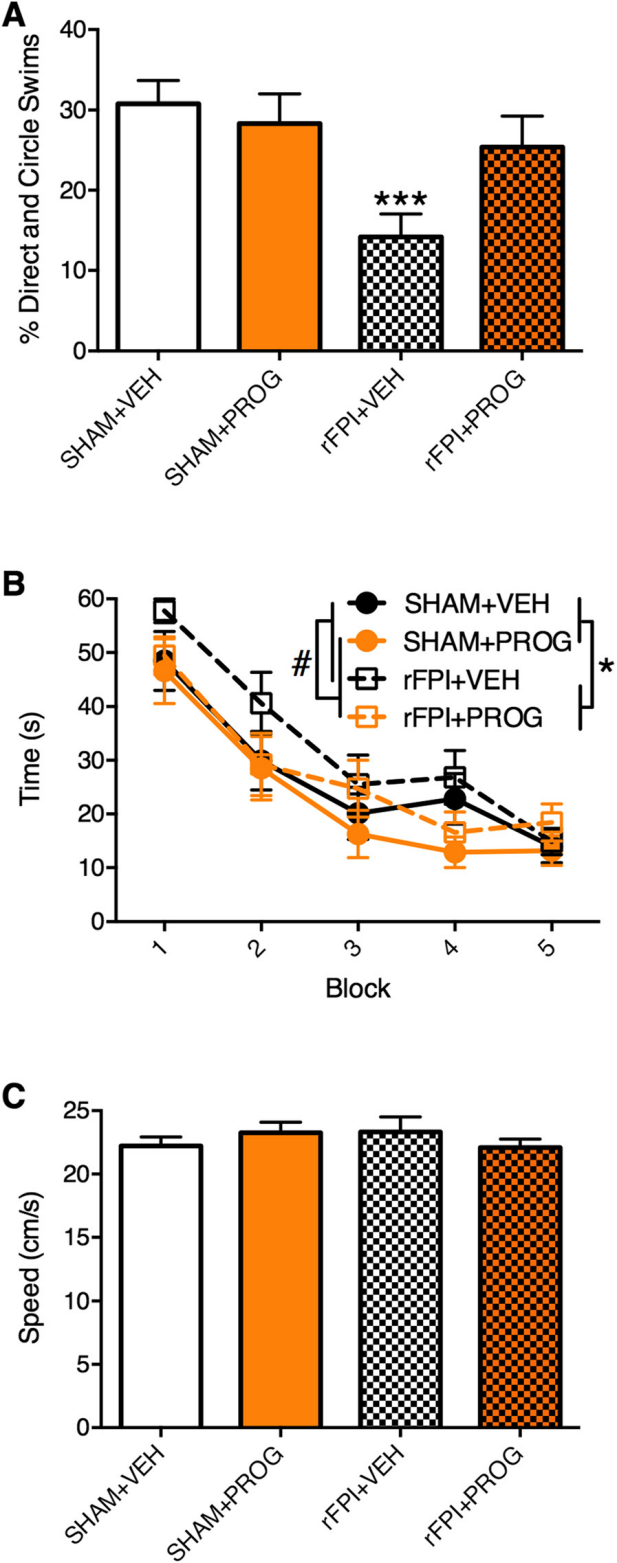
PROG treatment reduces cognitive deficits after rFPI. rFPI + VEH rats had significantly fewer direct and circle swims than all other groups during water maze testing (**a**). PROG-treated groups also had faster search times (**b**). There were no significant differences on the measure of swim speed (**c**), suggesting that motor deficits did not confound cognitive measures during water maze testing. *Triple asterisks* significantly different than all other groups, *number sign* significant treatment effect, *p* < 0.05

ANOVA indicated a significant injury × treatment interaction on the measure of direct and circle swims during water maze testing (*F*_1, 46_ = 4.133, *p* < 0.05, Fig. 1a). Post hoc analysis found that rFPI + VEH rats performed fewer direct and circle swims than all other groups (*p* < 0.05), whereas the rFPI + PROG group did not significantly differ from either of the SHAM groups. There was also a significant treatment effect found for the measure of search time (*F*_1, 46_ = 4.387, *p* < 0.05, Fig. 1b), indicating that rats treated with PROG located the platform faster than those treated with VEH. Significant effects for injury were also found for both direct and circle swims (*F*_1, 46_ = 8.474, *p* < 0.01) and search time (*F*_1, 46_ = 5.607, *p* < 0.05). There were no statistically significant differences on the measure of swim speed (Fig. 1c), suggesting that motor impairments did not confound the cognitive measures.

**Fig. 2:**
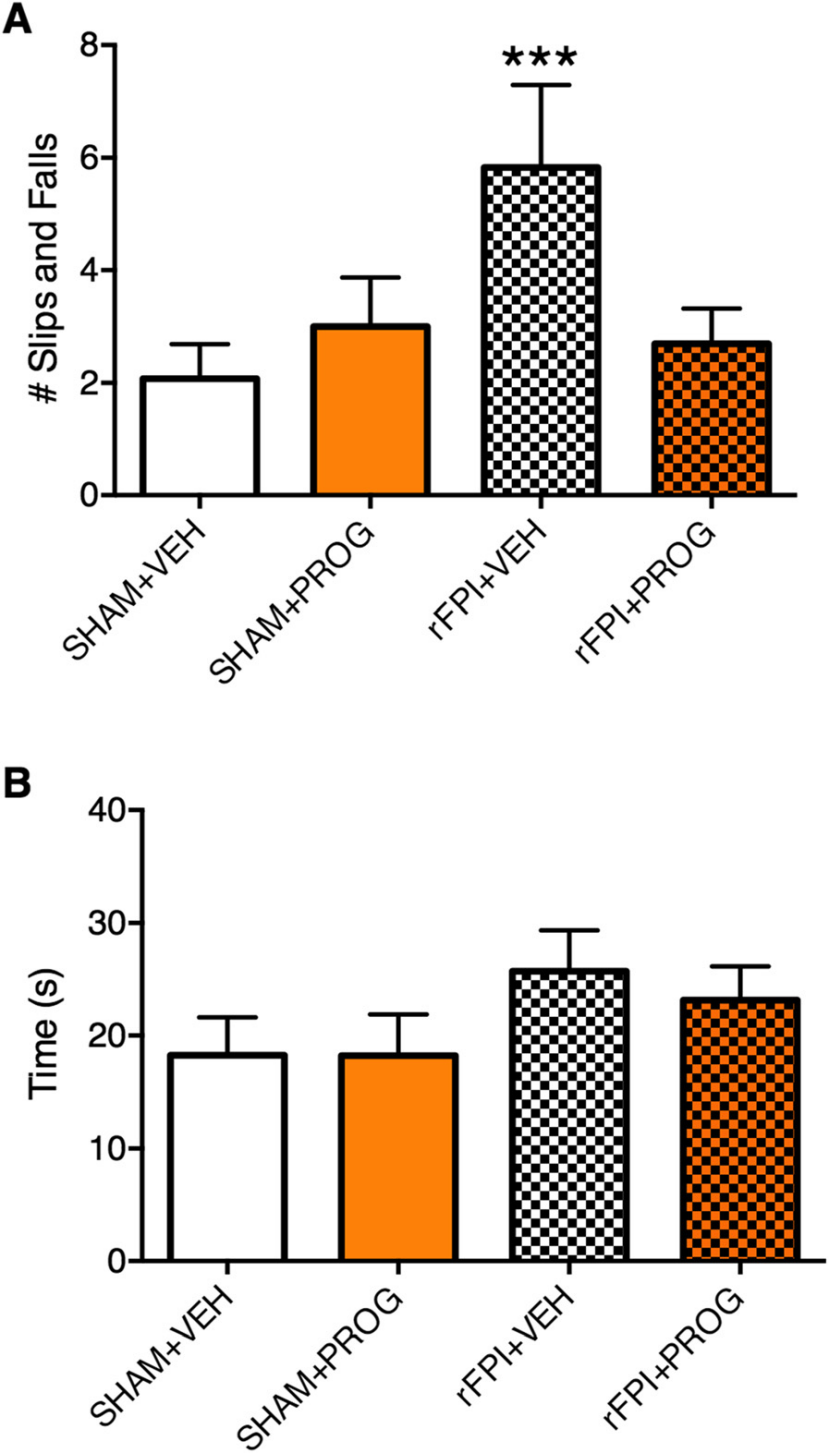
PROG treatment reduces motor deficits after rFPI. rFPI + VEH rats displayed more slips and falls compared to all other groups on the beam task (**a**). There were no significant differences on the measure of average traverse times (**b**). *Triple asterisks* significantly different from all other groups, *p* < 0.05

ANOVA revealed a significant injury × treatment interaction on the measure of slips and falls on the beam task (*F*_1, 46_ = 51.533, *p* < 0.05, Fig. 2a). Post hoc analysis indicated that the rFPI + VEH group had significantly more slips and falls than all other groups (*p* < 0.01), whilst the rFPI + PROG group did not significantly differ from either of the SHAM groups. There were no statistically significant findings on the measurement of average traverse times (Fig. 2b).

There were no statistically significant findings for the elevated plus maze, open field or forced swim task (all *p* > 0.05).

### PROG treatment reduces brain damage in rFPI

#### MRI volumetrics

**Fig. 3:**
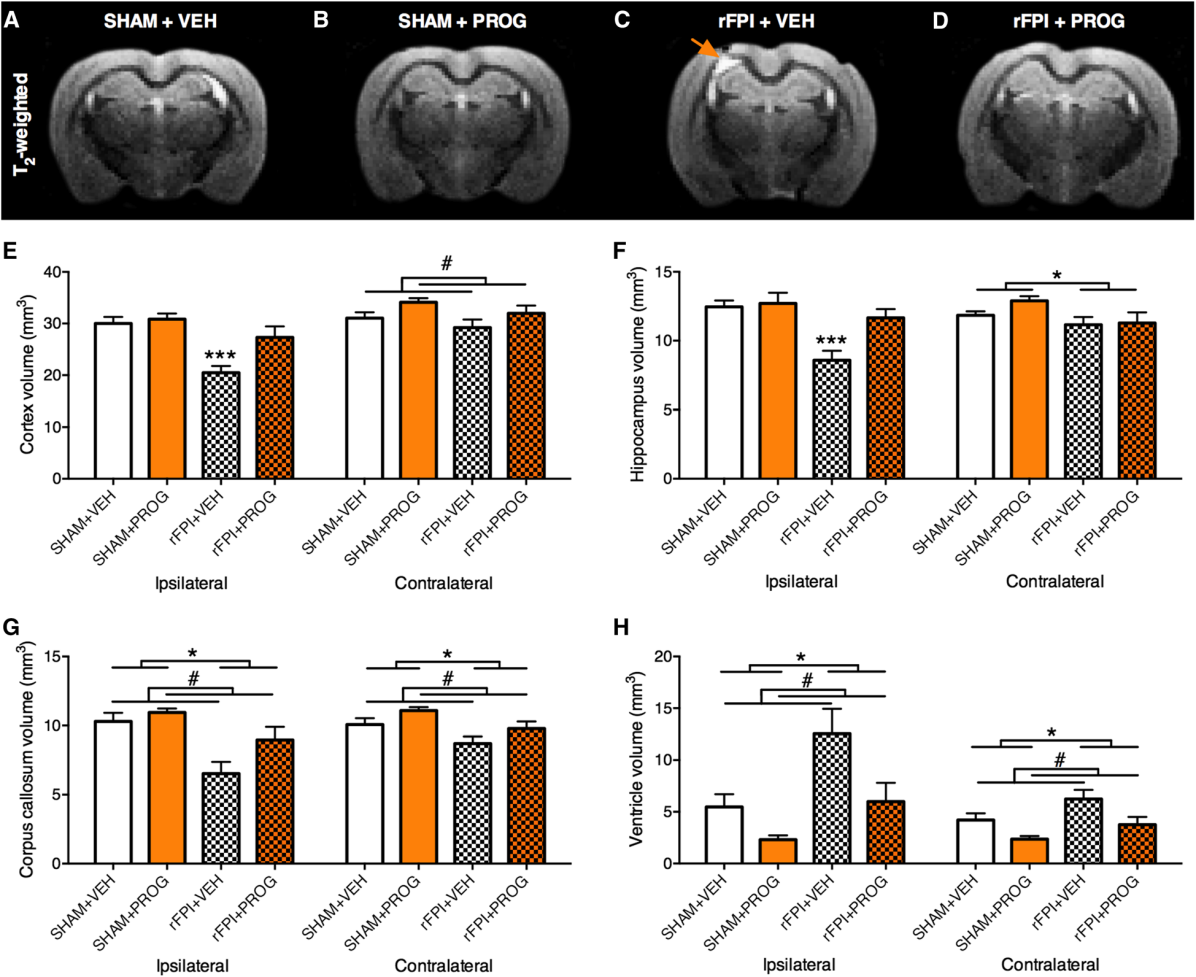
PROG treatment attenuates brain atrophy after rFPI. As shown in the representative T_2_-weighted MRI images (**a**–**d**), rFPI + VEH rats had significantly less volume of the ipsilateral cortex (**e**) and ipsilateral hippocampus (**f**) than all other groups, whereas the rFPI + PROG group did not significantly differ from SHAM groups. PROG-treated rats also had increased volume of the ipsilateral corpus callosum (**g**), contralateral corpus callosum (**g**) and contralateral cortex (**e**) and smaller lateral ventricles (**h**). Rats given rFPI had reduced volume of contralateral hippocampus (**f**), ipsilateral corpus callosum (**g**), contralateral corpus callosum (**g**) and larger lateral ventricles (**h**). *Triple asterisks* significantly different from all other groups, *number sign* significant treatment effect, *single asterisk* significant injury effect, *p* < 0.05

As shown in the representative MRI images (Fig. 3a–d), there was a loss of ipsilateral cortex and ipsilateral hippocampus volumes in rFPI + VEH rats as compared to all other groups. ANOVA revealed a significant injury × treatment interaction in the ipsilateral cortex (*F*_1, 26_ = 4.237, *p* < 0.05, Fig. 3e) and ipsilateral hippocampus (*F*_1, 26_ = 4.697, *p* < 0.05, Fig. 3f). Post hoc analysis indicated a significant reduction of ipsilateral cortex and ipsilateral hippocampus volumes in rFPI + VEH group compared to all other groups (*p* < 0.01), whereas the rFPI + PROG group did not differ significantly from either SHAM group.

Significant treatment effects were found in the ipsilateral corpus callosum (*F*_1, 26_ = 16.801, *p* < 0.001, Fig. 3g), contralateral cortex (*F*_1, 26_ = 5.357, *p* < 0.05, Fig. 3e), and contralateral corpus callosum (*F*_1, 26_ = 5.659, *p* < 0.05, Fig. 3g), indicating that PROG-treated rats had significantly more volume of these brain structures compared to VEH-treated rats. There were also significant treatment effects in the ipsilateral (*F*_1, 26_ = 9.626, *p* < 0.005, Fig. 3h) and contralateral lateral ventricles (*F*_1, 26_ = 10.687, *p* < 0.01, Fig. 3h), indicating that PROG-treated rats had smaller lateral ventricle volumes than rats treated with VEH.

Significant injury effects were found in the ipsilateral corpus callosum (*F*_1, 26_ = 4.887, *p* < 0.05), contralateral hippocampus (*F*_1,26_ = 5.190, *p* < 0.05), and contralateral corpus callosum (*F*_1, 26_ = 9.220, *p* < 0.005), indicating that rFPI rats had significantly less volume of these structures than SHAM rats. There were also significant injury effects in the ipsilateral (*F*_1, 26_ = 11.812, *p* < 0.005) and contralateral (*F*_1, 26_ = 6.727, *p* < 0.05) lateral ventricles indicating that rFPI rats had significantly larger lateral ventricles than SHAM rats.

#### DTI

**Fig. 4:**
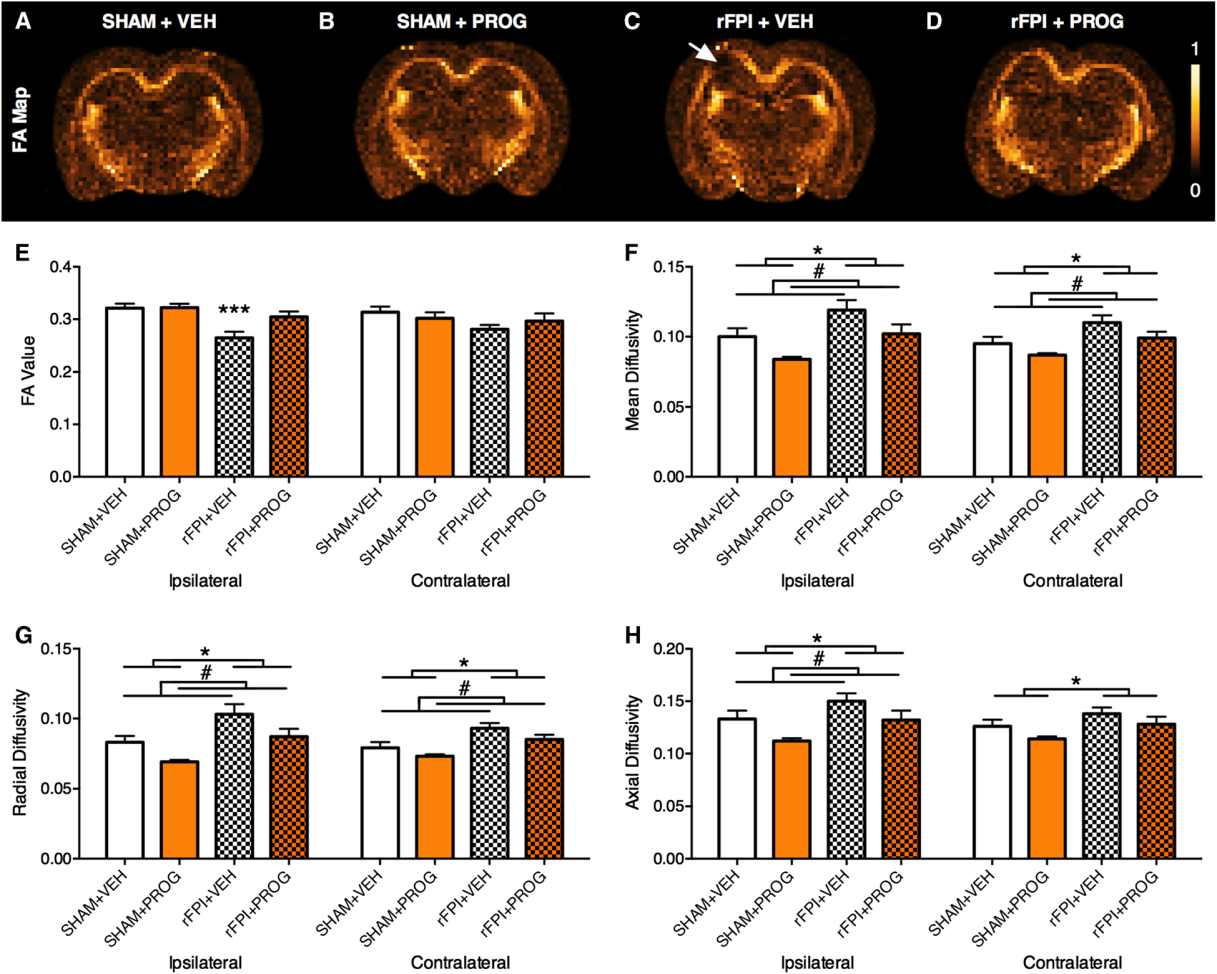
PROG treatment mitigates corpus callosum injury after rFPI. As shown in the representative fractional anisotropy (FA) maps (**a**–**d**), rFPI + VEH rats had significantly reduced mean FA in the corpus callosum (**e**) compared to all other groups, whereas the rFPI + PROG group did not significantly differ from SHAM groups. **f** PROG-treated rats had decreased mean diffusivity in the ipsilateral and contralateral corpus callosum, whereas rFPI rats had increased mean diffusivity in the ipsilateral and contralateral corpus callosum. **g** PROG-treated rats had decreased radial diffusivity in the ipsilateral and contralateral corpus callosum, whereas rFPI rats had increased radial diffusivity in the ipsilateral and contralateral corpus callosum. **h** PROG-treated rats had decreased axial diffusivity in the ipsilateral corpus callosum, whereas rFPI rats had increased axial diffusivity in the ipsilateral and contralateral corpus callosum. *Triple asterisks* significantly different than all other group, *number sign* significant treatment effect, *single asterisk* significant injury effect, *p* < 0.05

The integrity of the corpus callosum, a major white matter tract commonly affected by mTBI, was assessed using DTI. ANOVA found a significant injury × treatment interaction (*F*_1, 26_ = 4.340, *p* < 0.05, Fig. 4e) on the measure of FA in the ipsilateral corpus callosum. Post hoc analysis found that rFPI + VEH rats had a significant reduction in FA compared to all other groups (*p* < 0.01), suggesting axonal injury. Notably, rFPI + PROG rats were not significantly different than shams. There were no differences in FA findings in the contralateral corpus callosum.

In addition, significant treatment effects indicated that VEH-treated rats had increased mean diffusivity (ipsilateral corpus callosum, *F*_1, 26_ = 7.876, *p* < 0.005; contralateral corpus callosum, *F*_1, 26_ = 4.313, *p* < 0.05; Fig. 4f), radial diffusivity (ipsilateral corpus callosum, *F*_1, 26_ = 8.127, *p* < 0.01; contralateral corpus callosum, *F*_1, 26_ = 4.443, *p* < 0.05; Fig. 4g), and axial diffusivity (ipsilateral corpus callosum, *F*_1, 26_ = 6.897, *p* < 0.05, Fig. 4h). Significant injury effects further revealed that rats given rFPI also had increased mean diffusivity (ipsilateral corpus callosum, *F*_1, 26_ = 10.570, *p* < 0.005; contralateral corpus callosum, *F*_1, 26_ = 10.047, *p* < 0.005; Fig. 4f), radial diffusivity (ipsilateral corpus callosum, *F*_1, 26_ = 13.924, *p* < 0.001; contralateral corpus callosum, *F*_1, 26_ = 14.225, *p* < 0.001; Fig. 4g), and axial diffusivity (ipsilateral corpus callosum, *F*_1, 26_ = 6.001, *p* < 0.05; contralateral corpus callosum, *F*_1, 26_ = 5.124, *p* < 0.05; Fig. 4h).

### PROG treatment reduces neuroinflammation and oxidative stress in rFPI

**Fig. 5:**
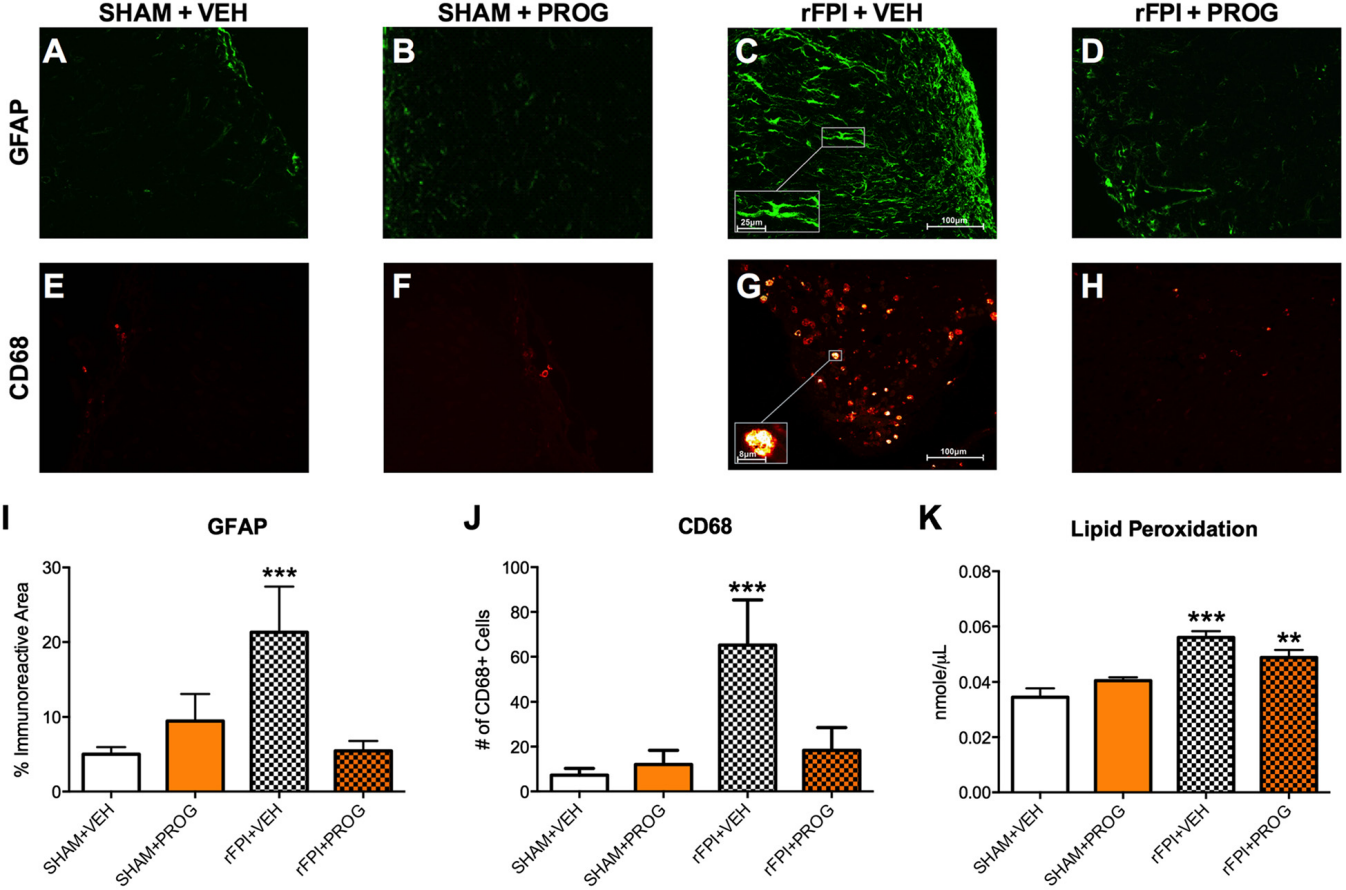
PROG treatment reduces neuroinflammation and oxidative stress after rFPI. As shown in the representative images of GFAP (**a**–**d**) and CD68 (**e**–**h**) immunofluorescence staining, rFPI + VEH rats had significantly increased GFAP immunoreactive area (**i**) and CD68-positive cells (**j**), whereas the rFPI + PROG group did not significantly differ from SHAM groups on either measure. The rFPI + VEH rats also showed significantly increased levels of MDA, a marker of lipid peroxidation, compared to all other groups (**k**). Though rFPI + PROG rats had significantly less lipid peroxidation than rFPI + VEH rats, they had significantly more lipid peroxidation than both SHAM groups. *Triple asterisks* significantly different than all other groups, *double asterisks* significantly different than SHAM groups, *p* < 0.05

There was a significant injury × treatment interaction for GFAP immunoreactive area in the injured cortex (*F*_1, 17_ = 7.642, *p* < 0.05, Fig. 5i). Post hoc analysis found that rFPI + VEH rats had a significant increase of GFAP compared to all other groups (*p* < 0.01), whereas there was no difference between rFPI + PROG and SHAM groups. As shown in Fig. 5c, rFPI + VEH rats had densely stained and hypertrophic astrocytes with long and overlapping processes. These findings suggest that treatment with PROG attenuated rFPI-induced reactive astrogliosis.

There was a significant injury × treatment interaction for the number of CD68-positive cells in the injured cortex (*F*_1, 17_ = 5.206, *p* < 0.05, Fig. 5j). Post hoc analysis found that rFPI + VEH rats had significantly more CD68-positive cells than all other groups (*p* < 0.05), whereas there was no difference between rFPI + PROG and SHAM groups. As shown in Fig. 5g, these CD68-postive cells had an amoeboid morphology typical of phagocytic macrophages. These findings suggest that treatment with PROG attenuated the rFPI-induced activation of macrophages/microglia.

ANOVA found a significant injury × treatment interaction (*F*_1, 13_ = 7.177, *p* < 0.05, Fig. 5k) in MDA, an indicator of lipid peroxidation. Post hoc analysis found that rFPI + VEH rats had significantly higher levels of MDA than all other groups, including the rFPI + PROG group (*p* < 0.05). However, it was also found that rFPI + PROG rats had significantly higher levels of MDA than both SHAM groups (*p* < 0.05), suggesting that treatment with PROG reduced rFPI-induced lipid peroxidation but did not prevent it.

## Discussion

Repetitive mild brain injuries can result in cumulative brain damage and neurodegeneration that has been linked to neuroinflammation and oxidative stress, and there are currently no clinically available treatment options known to mitigate these effects in rmTBI patients. Therefore, here, we conducted the first study to evaluate the potential of PROG to reduce neuroinflammation and oxidative stress, and the associated neurodegeneration and neurological impairments, in experimental rmTBI. Rats given rFPI and treated with VEH had increased neuroinflammation and oxidative stress, with concomitant damage to grey and white matter brain structures, and cognitive and motor deficits at 12 weeks post-injury. Importantly, rats given rFPI and treated with PROG had significantly less neuroinflammation, oxidative stress and brain damage, in addition to improved cognitive and sensorimotor function compared to their VEH-treated counterparts. These novel data demonstrate that PROG treatment has anti-inflammatory, anti-oxidant and neuroprotective effects in experimental rmTBI and thus presents potential as a therapeutic intervention for the clinical presentation of this syndrome.

### The role of neuroinflammation and oxidative stress in rmTBI

The pathogenesis of rmTBI likely involves a number of secondary injury processes. Growing evidence, supported by our findings here, implicates neuroinflammation and oxidative stress as key contributing factors [7, 8]. Sustained neuroinflammation, characterized by activation of microglia and astrocytes, is known to be a key mediator of progressive secondary injury after brain insult and other neurodegenerative conditions [8]. With regard to mTBI, we and others have reported transient neuroinflammation after a single mild FPI in rats that becomes exacerbated and sustained upon repeated insults [10–12, 23, 25, 30]. Consistent with these findings, we observed astrogliosis, as indicated by increased GFAP immunoreactivity, and increased activated microglia and macrophages, as evidenced by increased CD68-positive cells with amoeboid morphologies, in the injured cortex of rFPI + VEH rats at 12 weeks post-injury.

Once activated, astrocytes and microglia undergo morphological changes. Reactive astrocytosis involves cellular hypertrophy, lengthened processes and increased expression of GFAP [35]. Activated microglia undergo a morphological change, taking on an amoeboid shape resembling peripheral macrophages, and scavenge the damaged CNS, performing phagocytic functions [39, 40]. Activated astrocytes and microglia produce and release a number of pro-inflammatory mediators, including the cytokine tumour necrosis factor-alpha (TNF-α) and interleukin-1 beta (IL-1β) that may contribute to secondary brain damage through mechanisms such as apoptosis [39, 40]. Activated microglia/macrophages, reactive astrocytes and other immune cells can also produce and release reactive oxygen species, such as nitric oxide, that contribute to oxidative stress—an imbalance of reactive oxygen species and endogenous anti-oxidants agents [11, 28, 39–43]. Neuroinflammatory factors such as NF-κB, signaling downstream of TNF-α and nitric oxide, are robustly elevated after TBI and likely contribute to oxidative stress and neuronal loss, which in turn further exacerbate the inflammatory response [44, 45]. One consequence of oxidative stress is lipid peroxidation, which involves the oxidative degradation of lipids in cell membranes [11, 21, 28, 46]. We also detected an increase in lipid peroxidation in rats given rFPI + VEH.

Our findings of persisting neuroinflammation and oxidative stress occurred in the presence of structural brain damage and functional impairments, which suggests a role for these secondary injury pathways in the pathogenesis of rmTBI. Further supporting this notion, we found that PROG attenuated the persisting neuroinflammation and oxidative stress and that these changes were associated with decreased brain damage and improved long-term cognitive and motor outcomes in rats given rmTBI. These results of anti-inflammatory and anti-oxidant effects of PROG treatment in rmTBI are consistent with previous experiments in moderate to severe TBI studies [19–22]. Whilst the exact mechanisms remain unclear, PROG is thought to act on inflammation and oxidative stress both directly [47, 48] and indirectly by the modulation of toll-like receptors and NF-κB signaling with a concurrent reduction in cytokines [49, 50]. Nitric oxide has also been shown to be reduced by post-injury treatment with PROG, possibly through the prevention of inducible nitric oxide synthase, an isoform that abundantly produces nitric oxide during secondary injury [51, 52]. It should be noted that although rFPI + PROG rats had significantly reduced levels of lipid peroxidation than the rFPI + VEH group, MDA levels were still elevated compared to both SHAM groups. This remaining oxidative stress may have contributed to the relatively mild MRI-detected brain damage that was still evident in the rFPI + PROG rats. That PROG treatment was not able to fully negate oxidative stress may be due to a number of reasons, including dose and duration of the PROG treatment or the potential contribution of other mechanisms not targeted by PROG, and requires further exploration. For example, future experimentation could look at a combinatorial therapy, with the addition of agents targeting other secondary pathways.

### The nature of cognitive and motor deficits after rmTBI

Spatial cognition, as assessed in the water maze [53, 54], was shown to be impaired in rFPI + VEH rats through the measure of direct and circle swims, consistent with previous data [10, 11]. Direct and circle swims represent the most efficient route to a known escape position in the water maze, indicating that rFPI + VEH rats were less efficient at the task than the other groups [10, 11, 53, 54]. As there were no group differences on the measure of swim speed during water maze testing, or any anxiety-related outcomes in other tasks, the deficits observed in the rFPI + VEH rats were likely cognitive in nature. Notably, the rFPI + PROG group did not significantly differ from the two SHAM groups on the measure of direct and circle swims, suggesting that PROG was able to preserve cognitive function after rFPI. There was also a significant treatment effect indicating that PROG-treated rats located the hidden platform faster than VEH-treated rats, which further supports the notion that PROG preserved cognitive function in the water maze.

Consistent with previous studies [10, 11], the rFPI + VEH rats displayed significant sensorimotor deficits, as indicated by more slips and falls on the beam task compared to all other groups [55]. In contrast, the rFPI + PROG rats did not show any significant sensorimotor deficits relative to the SHAM groups. As there were no differences found in average beam traverse times, nor in other gross motor measures from the other behavioural tasks between groups, these sensorimotor deficits occurring in the rFPI + VEH rats was likely mild in nature. Nonetheless, these findings indicate that PROG treatment is able to attenuate both sensorimotor and cognitive dysfunction after rFPI.

The cognitive and sensorimotor deficits in the rFPI + VEH rats, and the preservation of these functions in the rFPI + PROG rats, may be related to the degree of brain damage in these animals. MRI-based volumetric analysis of the ipsilateral cortex and ipsilateral hippocampus revealed significant atrophy of these structures in the rFPI + VEH rats that did not occur in the rFPI + PROG rats. Of particular note, the sensorimotor cortex is situated near the FPI impact area [27, 56, 57], and damage to the cortex and hippocampus has been linked to cognitive and sensorimotor deficits in TBI [27, 58–61]. DTI analyses of the corpus callosum, a major white matter tract commonly affected by TBI [62], showed significant diffusion abnormalities suggestive of axonal injury in the rFPI + VEH group but not in the rFPI + PROG group. Finally, a previous study found that rats given a TBI had increased cell proliferation and survival of immature neurons in the dentate gyrus of the hippocampus and that PROG treatment normalized these effects [63]. Because the hippocampus is an important structure involved in cognitive function, it is interesting to speculate whether abnormalities in neurogenesis may be involved in the cognitive outcomes we found here. Taken together, it is likely that changes to each of the abovementioned brain structures may have contributed to the behavioural findings in this study. However, further research is required to determine the precise mechanisms underlying the functional deficits occurring in rmTBI.

### PROG as a treatment for mTBI patients?

To our knowledge, this is the first study to demonstrate that PROG reduces neuroinflammation, oxidative stress, brain damage and functional deficits in experimental rmTBI, particularly in the more chronic stage of injury. Considering that there is currently no effective treatment known to reduce the detrimental long-term effects caused by rmTBI, these findings hold important implications for the future treatment of mTBI patients. In this study, we began PROG treatment after the first mTBI and continued treatment until 5 days after the third injury. As such, PROG treatment was given prophylactically for the second and third injuries, and this may have contributed to the protective effects. In the clinical setting, this could be comparable to a high-risk individual (e.g., athlete or soldier) who has already suffered one mTBI and continues treatment for the remaining duration of their high-risk activity (e.g., season or tour of duty). As the recommended time window for PROG treatment after a brain insult is within 2 h [64, 65], this preventative treatment paradigm may be advantageous within these high-risk environments. However, there are other possible scenarios where PROG treatment may be beneficial as a therapy for mTBI. For example, it is possible that PROG treatment acutely after a single mTBI may improve recovery, prevent post-concussion syndrome or reduce vulnerability to a second brain insult. PROG treatment in the chronic stages post-mTBI may also be beneficial for the minority of mTBI patients who do suffer a persisting post-concussion syndrome or for individuals who have suffered past mTBIs and have persisting neuroinflammation. Of particular interest, a recent study utilizing PET imaging found that retired NFL players with a history of mTBI had increased binding of [^11^C]DPA-713 to translocator protein, an indicator of neuroinflammation, as well as concomitant brain atrophy [66]. Therefore, treatment with PROG at chronic stages post-injury should also be explored, and the use of neuroinflammatory biomarkers (e.g., PET) could allow for a more precise medical approach. Taken together, there are a number of clinical mTBI settings where PROG treatment may be applicable; however, future studies are clearly required to investigate whether PROG treatment is beneficial in these various scenarios and to determine the optimal therapeutic windows and dose.

Importantly, PROG has a long history of clinical use and safe administration in both male and female patients [67, 68]. This includes recent phase III clinical trials with PROG treatment in severe TBI patients [69, 70]. Unfortunately, these trials did not show improvement on the Glascow Outcome Scale and Extended Glascow Outcome Scale at 3 and 6 months post-injury in moderate and severe TBI patients treated acutely with PROG [69, 70]. Though these trials failed to demonstrate efficacy for PROG treatment in more severe TBI, the authors of these studies raised a number of limitations that are important to consider including limited/insensitive outcome measures, unidimensional TBI characterization approaches (e.g., Glascow coma scale) and the heterogeneity of severe TBI [69–73]. It has also been suggested that sub-optimal doses were used in the PROG trials [71–73], which highlight the need to measure PROG levels in future clinical and pre-clinical studies. It is also worth speculating whether PROG, which targets secondary injury processes, may be better suited to attenuate responses in the more subtle pathological environment occurring in mTBI and rmTBI, where there is little to no irreversible primary injury. This is in contrast to moderate and severe TBI, where the primary injury incurred at the moment of impact contributes to a large part of the deficits seen in patients, and initiates a robust secondary injury response resulting in considerable swelling, cell death and tissue loss [74].

## Conclusions

Here, we examined whether PROG treatment in rats given rmTBI improved long-term outcomes. Progesterone treatment significantly attenuated markers of neuroinflammation and oxidative stress in rats given rmTBI, with concomitant reductions in behavioural impairments and brain damage. These findings further implicate neuroinflammation and oxidative stress in the pathophysiological aftermath of mild brain injuries. Furthermore, the proven safety and limited negative side effects of PROG, along with our findings here, suggest that PROG may be a viable treatment option within the context of mTBIs and their more chronic consequences.

## Abbreviations

ANOVA: analysis of variance
CTE: chronic traumatic encephalopathy
DTI: diffusion tensor imaging
FA: fractional anisotropy
MDA: malondialdehyde
mTBI: mild traumatic brain injury
PROG: progesterone
rFPI: repeated mild fluid percussion injury
rmTBI: repeated mild traumatic brain injuries
ROI: regions of interest
SHAM: sham injury
TBARS: thiobarbituric acid reactive substances
VEH: cyclodextrin vehicle
